# Evaluation of a fluorosis prevention educational program: A randomized field trial

**DOI:** 10.4317/jced.54225

**Published:** 2018-05-01

**Authors:** Fatima-Carmen Aguilar-Díaz, Ma Esther Irigoyen-Camacho, Socorro-Aída Borges-Yáñez

**Affiliations:** 1 PhD, National Autonomous University of Mexico. School of Higher Education, campus León. Public Health Department; 2 PhD, Metropolitan Autonomous University. Health Care Department; 3 PhD, National Autonomous University of Mexico. Dental Public Health Department. Graduate Division,School of Dentistry

## Abstract

**Background:**

A 2-group randomized field trial was conducted to evaluate the impact of a fluorosis educational preventive program in mother´s knowledge and practices, and on the urine fluoride concentration of their preschool children.

**Material and Methods:**

A group of 139 mother-child pairs participated in the study. Randomly, children were assigned to an intervention group, their mothers were participants of an educational program, or a control group (CG); including 69 and 70 child-mother pairs, respectively, the follow-up period was six months. Mother´s knowledge and practices were evaluated and children´s first urine sample was used to measure fluoride concentration at the beginning of the study and at the end of the follow-up period.

**Results:**

The mean age of the children was 4.18 (sd 0.62) years-old at baseline. Mothers in the IG improved their knowledge and practices associated with fluorosis risk factors. Adequate knowledge about the amount of toothpaste to use for brushing improved in the IG (*p*=0.006). In 82.1% of the children in the IG showed decrease in urine fluoride concentration was observed (*p*< 0.001), no significant differences were shown in the CG.

**Conclusions:**

Mothers participating in an education program improved their knowledge and practices, reducing the risk of dental fluorosis in their children who showed a decreased on their urine F concentration.

** Key words:**Knowledge, practices, urinary fluoride, water fluoride, preschool children, mothers.

## Introduction

Dental fluorosis (DF) is a developmental disturbance of hard tooth tissues, caused by excessive exposure to fluoride during tooth development affecting its organization, composition and structure. In high dosages fluorides produce a lower dental mineral content and increased porosity in the enamel and dentin (1,2). In the world, at least 25 countries suffer from endemic fluorosis, only in China 26 million people present fluorosis (3).

Fluoride is nowadays widely available through artificially fluoridated products as mouth rinses, toothpastes and other fluoridated products, and used as part of public health programs for dental caries control, such as drinking water, table-salt and milk, which have demonstrated efficacy (4). Nonetheless, multiple availability, widespread use and/or misuse of fluoridated products at the same time have contributed to increase DF prevalence (5,6). One of these practices of misuse is swallowing fluoridated toothpaste, as happens with children less than five years old (7).

Consumption of water or bottled beverages such as some juices, soft drinks or carbonated fruit drinks with high concentrations of fluoride is another risk factor of DF (8,9). In regions where the fluoride content in tap water varied between 1.5-2,0 mg/L people are 4.4 times more likely to develop DF than in areas with lower fluoride concentration (10). Using this water for cooking purposes increases prevalence and severity of fluorosis (11), as boiling the water results in an increased of fluoride by up to 66% (12). The use of milk formulas reconstituted with tap water also may increase the risk of DF (13).

There have been efforts to prevent DF as placing water filters to decrease concentration of fluoride (F), or seeking safer water sources, in areas with naturally high water fluoride concentration. However, it has been suggested that these strategies should be accompanied by educational programs to avoid fluorosis risk factors (14). Trough empowerment, conceptualized as a framework for understanding the process and consequences of efforts to exert control and influence over the decisions that affect one’s life, including perceptions of personal control and behaviors (15). Empowering mothers, providing adequate information that allows them developing good practices to avoid excessive consumption of fluoride in their young children is important.

Nowadays there is little information on the effect of educational programs intended to help mothers of preschool children to recognize and avoid fluorosis risk factors in endemic fluorosis areas. The aim of this study was to evaluate the impact of a fluorosis prevention educational program in mother’s knowledge and practices, and on the fluoride concentration in urine of their preschool children. We hypostatized that mother-child in the intervention group (IG) would have better knowledge, would report less risk practices and their children would show lower urine fluoride concentration than Control Group participants.

## Material and Methods

A 2-group randomized field trial of 6 months of follow up was performed on mothers and their children, selected from a kindergarten in a community southeast of Morelos, Mexico. This is a community with a medium level of marginalization, 41.85% of population aged 15 years or more has not completed elementary education level, and 9.7% inhabited dwellings with dirt floor. Concentration of F in groundwater of 1.6 ppm has been reported (16). Eligible participants were children born and remained in the study area.

To calculate the sample size required for the study a 50% prevalence of high concentration of fluoride in urine was assumed and a 25% decrease in this prevalence was hypostatized in the IG, β= 0.20 α= 0.05 was used. Also, a 10% drop out rate was assumed. Evaluated variables were age, sex, mother’s knowledge, perception and risk practices regarding fluorosis and children´s urine fluoride concentration. Also, data about early infant feeding practices as habits of bottle feeding was recollected. Birth weight, age until which mothers fed their children with breast milk, no and/or with bottle was also registered.

The study was performed in two stages, and to assign every mother-child into a group, it was used the number of the school list children to randomly assigned to the CG or IG using a table of random numbers. In the first stage, we worked with the CG, recollecting data and urine samples. In the second stage, the fluorosis educational program was implemented through a series of scheduled meetings with the IG. The first three meetings were held on a weekly basis, then reinforcements at weeks 4, 8 and 12 were performed. Educational sessions were achieved through a series of talks aimed at the parents improving their ability to avoid risk practices, including educational content about the condition (i.e. What is DF, causes, implications, risk factors). They were trained in preventive measures as identifying and using salt without fluoride, to identify toothpaste fluoride contain, to use toothpaste properly, to avoid use of tap water to drink or cook, and to improve eating habits (i.e. Adequate intake of calcium, vitamin C and D and minerals). Parents were free to ask questions at any time during the sessions. Every session had a duration of approximately 40 minutes, 20 minutes were of oral presentation of the topic, supported by audiovisual material, 10 minutes of reinforcement with a didactic activity and 10 minutes of discussion-review doubts. Finally, pamphlets containing key information were provided. Samples of low-fluoride concentration dentifrice for each child were provided during the six-month period.

Children´s urine samples were collected in polyethylene containers (17), and fluoride concentrations were assessed at baseline and after six months in both study groups. Parents were asked to collect their child´s urinary samples to school. Instructions and containers were provided to perform this task, asking them to recollect the first morning voided urine of their children and bring the container to the school where it was gathered. The samples were kept at -20°C until they were analyzed directly by using a fluoride ion specific combination fluoride electrode (Orion # 4 star) and 25 Orion pH / Ion Meter (Orion). Analyses were carried out at the Autonomous University of Mexico. The lab technician who performed the fluoride quantification was blinded in relation to the origin of the sample, the CG or the IG. Clinical data about dental fluorosis on the children was recollected employing the Dental Fluorosis for Primary Dentition Index (DFPDI) (18), which stipulates that all teeth should be examined. This evaluation was performed by one examiner, a trained pediatric dentist, who was previously standardized, obtaining and intraexaminer kappa value of 0.82 for DFPDI.

This study followed the ethical and scientific standards developed to conduct biomedical research involving human beings, and established national and international guidelines. Parents or guardians signed a written consent and were aware that they could terminate their participation at any time. All results were managed to ensure the protection of individual rights and maintaining confidentiality. At the end of the study the intervention was provided to the CG. Ethical approval was obtained by the Ethical Committee of the Faculty of Dentistry of the National Autonomous University of Mexico.

-Statistical Analysis

Per-protocol analysis was carried out. For comparisons between IG and CG at base line, X2 test and t-Student test were applied for categorical and continuous variables, respectively. Considering the design of the study, generalized estimating equations model (GEE) using the Gauss family distribution, identity as link function and autoregressive correlation were applied to compare the mean of F urine concentrations between the IG and the CG. A log transformation of the fluoride concentration in urine was used as dependent variable for model construction to improve the distribution of this variable. The (exp(b) – 1)*100 was applied for regression coefficient interpretation. The significance level was set at *p*< 0.05.

## Results

**Figure 1:**
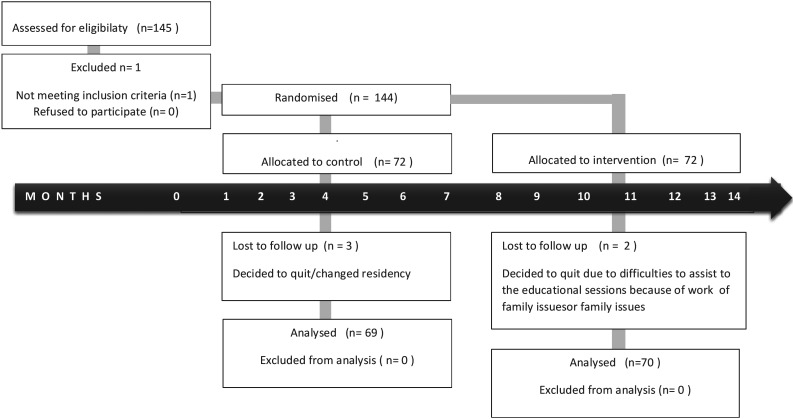
Participants flow chart.

A total of 145 mother-child pairs were included to participate, but during the follow up 6 pairs were missing. Final sample was 139 mother-child pairs, (dropout rate 3.5%) (Fig. 1), who were followed for a six-month period, 69 in the IG and 70 in the CG. Mother’s mean age was 28.4 (sd 5.4), children´s mean age was 4.18 (sd 0.62) years at baseline. There were no statistical significant differences in these two variables between the IG and the CG (*p*>0.05). In addition, no significant difference was detected in the percentage of boys and girls between these groups, educational level or occupational mother´s status; most of them had attended middle-school and were housekeepers ( Table 1).

**Table 1:**
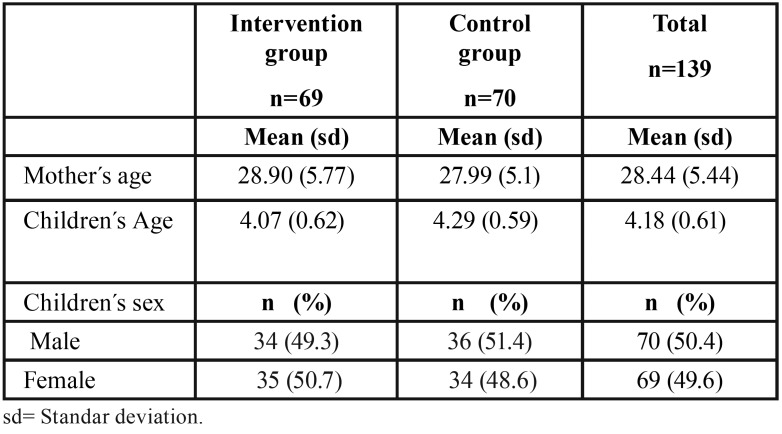
Children and mothers age and sex distribution in the intervention and in the control groups.

**Figure 2:**
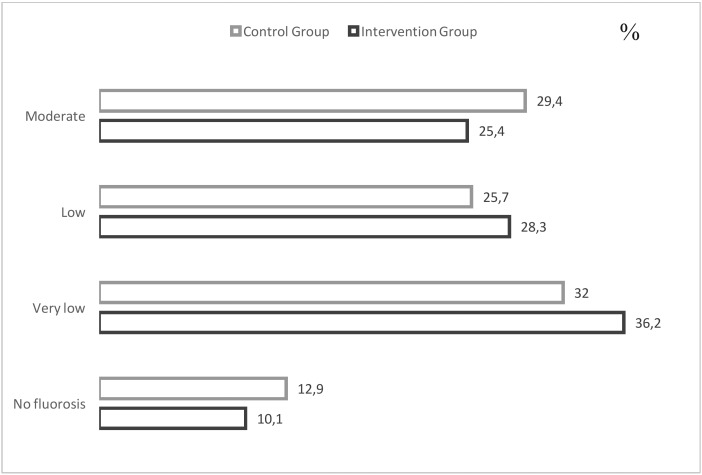
Dental fluorosis distribution among groups.

Dental fluorosis prevalence in molars of all preschoolers included was 88.5%; very mild dental fluorosis was observed in 29.5%, mild 25.9%, moderate in 25.9% and no severe cases were recorded (Fig. 2). No significant differences were identified in DF prevalence or severity between the two groups included (*p*>0.05).

We evaluated whether there was an association between the concentrations of F in urine with dental fluorosis. Kruskal-Wallis test was used to compared the mean F-concentration according to the severity of dental fluorosis and statistically significant differences were observed (Chi = 9.95, *p* = 0.019).

Data about early infant feeding practices as habits of bottle feeding was recollected, the distribution of these aspects did not show differences between groups. Birth weight of all children included was 3.050 kg, the average age until which mothers fed their children with breast milk was 15.02 months and 22.03 months with bottle, no differences were observed among study groups. Besides, these variables were evaluated according fluorosis severity and only the age until the child was bottle feeding was associated with DF severity, (*p*<0.001).

No significant differences were observed in knowledge about DF as have heard of the term “dental fluorosis”, the identification of products containing fluoride, etc., among the study groups at baseline data. Nonetheless after six months in the IG knowledge was better, significant differences were observed, 69.6% of the mothers of this group indicated to identify the cause of DF and 76.8% of the mothers considered their child to be at risk of DF. No significant differences were observed in the CG ( Table 2).

**Table 2:**
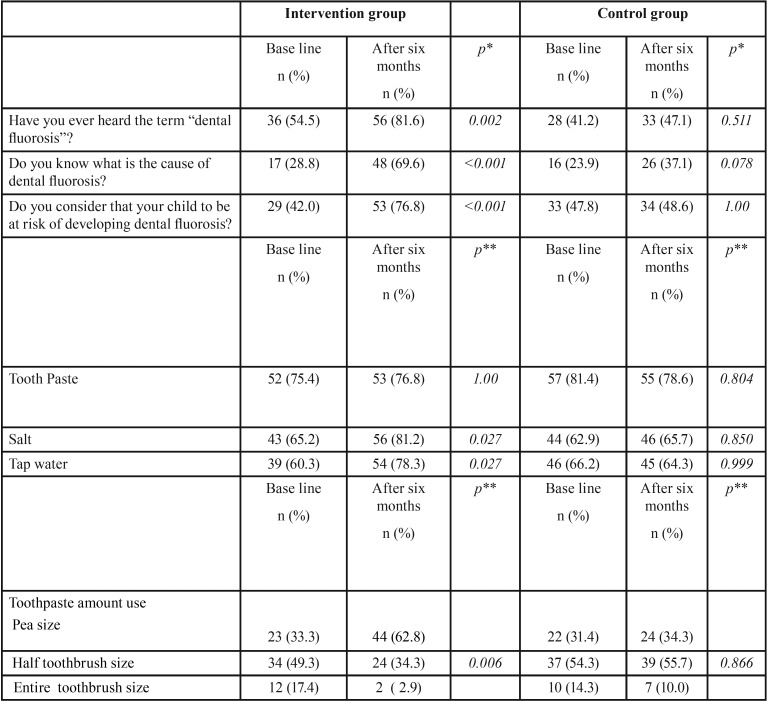
Mothers´ awareness, risk perception of dental fluorosis, identification of products containing fluoride and toothpaste use at baseline and six months later.

Mothers were asked to indicate from a list of products the ones they considered to contain fluoride. An increase was observed in the percentage of mothers in IG who identified table salt and tap water as sources of fluoride. A reduction in the amount of toothpaste was reported by the mothers in the IG, (*p*=0.006). Also in the IG, mothers reported less use of tap water to cook after the intervention ( Table 2).

Mean urine fluoride concentration among the entire sample was 1.77 mg/L (sd 0.87), no significant differences were found between groups at baseline. Fluoride concentration in urine in the IG decreased after six months, these differences were significant (*p*<0.001), ( Table 3). In 82.1% of the children in the IG a decrease in urine fluoride concentration was observed. In the CG the opposite effect was detected, at baseline fluoride concentration in urine was lower than after six months.

**Table 3:**
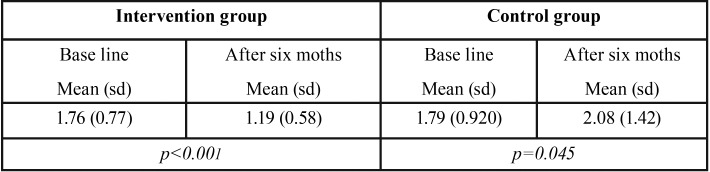
Mean Urine fluoride concentration at baseline and six months later in the intervention and the control group.

Results of the GEE model showed that in the IG the mean concentration of F in urine decreased by -0.22, 95% CI (-0.099 -0.394), controlled by sociodemographic variables as the age and sex of the children due to these have been linked to fluoride exposure and excretion. Transforming the scale of urinary concentration, it was found that children in the IG had a 19.7% lower fluoride concentration in urine than the children in the CG ( Table 4).

**Table 4:**
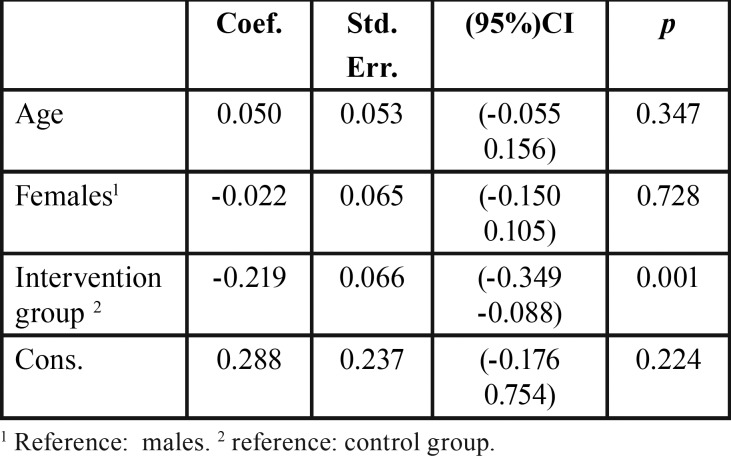
Factors associated to urine F concentration.

## Discussion

Positive results have been documented after implementing educational programs for diverse oral health conditions, improving knowledge and health practices but there are a few studies documenting the impact of these programs regarding DF (19). Dental fluorosis has a specific period of risk and so a specific period to prevent it and one way is improving knowledge in order to avoid risk practices. In this study, we observed improvements in mothers’ knowledge related to DF. Mothers modified their choices and changed their health behaviors; also, they changed their perception about their children being or not at risk of developing DF and the belief that taking actions would prevent it. More mothers in the IG recognized their children to be at risk to develop DF which probably promoted to take preventive actions as this acknowledgement is linked to behavior and the processes of decision-making and it is recognized as a key factor in modifying attitudes and health behaviors. In addition, we also observed a positive impact in certain risk practices, such as avoiding the use of tap water to cook and controlling the amount of toothpaste used, actions that would decrease the risk of DF.

Surprisingly, less than half of mothers have heard of the term “dental fluorosis”; notwithstanding that they live in an area of endemic fluorosis, also a small percentage of them identified the causes of this condition. Almost three-quarters of the total sample correctly identified that toothpaste contains fluoride, even before the intervention; nonetheless correct use of this product was low. It is necessary to mention that in Mexico pediatric formulated fluoride toothpaste is difficult to obtain and it is more expensive than regular toothpaste, therefore it is not widely used. The most common toothpastes sell in these communities have close to 1500 ppm F and so those are used by children since very young age when they have not the appropriate skills to spit out. This situation highlights the need to educate parents regarding the adequate selection and use of toothpaste. It would be important further evaluation of this educational program is assessing more objectively the total amount of tooth paste used.

Reduction in fluoride urine excretion was observed in those children whose mothers were trained in correct use of pediatric formulated toothpaste, emphasizing the use of a pea-sized amount of toothpaste. These results might lead to lower fluorosis prevalence or severity in the teeth that were still developing in these kindergarten children, as de Moura *et al.* showed in a follow up study that children whose parents were trained in the proper amount of fluoridated toothpaste used develop a lower incidence of DF (20).

Urine fluoride concentration was used instead of salivary because it is a better indicator of fluoride intake, while salivary concentration appears to be a better indicator of recent exposure to topical fluoride (21,22). Of the total fluoride intake in children aged 3-6 years 32–80% is excreted in urine (23). In children 3-5 years daily values of urinary excretion of fluoride considered to be optimal range from 0.36 to 0.48 mg F/day (23). In this study mean urine fluoride concentration was 1.77 mg F/l which is higher than that considered optimal and it is also higher to that reported in Jamaican children aged 2–6 years old (24). In Jamaica, as in Mexico, exists a salt fluoridated program (which stipulates the importance of regularly evaluating urinary fluoride concentrations). According to this program, in Mexico two kinds of table salts are manufactured, one iodized and another iodized and fluoridated. Salt distribution depends on water fluoride concentration; iodized-fluoridated1salt should not be distributed in those areas where natural water fluoride contain is higher than 0.7 ppm. The Mexican community where this study was performed belongs to Morelos and according to the law in this State it should only be distributed iodized-fluoridated salt. Nonetheless some studies suggest that in some areas inside this State, there are regions where water excides 0.7 ppm of fluoride so iodized salt should be distributed there. However most of the salt sell in that community is fluoridated, therefore it is particularly important to educate the community in this respect.

Regarding caries experience The National Center for Preventive Programs and Disease Control reports in 2011 for Morelos State a mean of DMF-t of 2.7-44 for children age 12 years. According to García-Pérez *et al.*, (25) who performed an evaluation in the same area of Morelos that the one evaluated in this study, reported a mean D3MFT and 0.61 (±1.47) in children with a mean age of 9.9 years, and they concluded that fluorosis at moderate and severe levels was associated with a higher prevalence of dental caries.

Fluoride exposure and F concentration in urine is directly related to fluoride intake (26-28). This association is affected by age, diet, fluoride used, among other factors, nonetheless it is generally accepted that urinary fluoride concentration is the best indicator of fluoride exposure, as it reflects the amount of fluoride ingested. It is assumed that in this study the F ingestion diminished in those children of the IG, who showed lower fluoride concentration. F decrement in urine may come from stopping the use of adult formulated fluoridated toothpaste and because of mothers cooked less frequently with fluoridated salt and tap water.

To control fluoride exposure is complex, due to the increase in the availability of sources of fluoride, and this is more difficult in areas where water fluoride concentration is high, as in the community studied, where risk increases due to the combination of sources. This underscores the importance of educating parents about preventive measures that are within its power to prevent dental fluorosis. It would be suggested to perform this intervention in mothers of younger children to obtain more benefits in specific in the prevalence and severity of DF fluorosis in the anterior teeth. The study has several limitations. It was not possible to evaluate the amount of fluoride ingested by various food and beverage products, the urinary sample was collected only in the morning and it was not possible to obtain other samples over a 24 hours period, nonetheless several studies have used such samples because of the difficulties involved in collecting 24 hours samples, and the results seem to be adequate (28), even more considering that the purpose of the study was to detect differences rather than the total amount of fluoride excreted. Some of the strengths of the study include its longitudinal design and the low dropout rate. It is suggested to continue with the evaluation of this educational program to gather further evidence including other variables and in longer periods.

It is pertinent to mention that our results do not tend to diminish the widely accepted and proved anti-caries effect of the water, salt and toothpaste fluoridation (4,29) but to emphasize the importance of not adding two or more sources of “systemic” fluoride because this action could increase the risk of fluorosis and with our intervention we attempt to avoid this situation. Even more we believe that our intervention would also provide benefits in communities where optimal water fluoridation exists, and no additional sources of fluoride are needed.

In conclusion, this education program improved knowledge and practices to reduce risk of DF. Urine F concentration decreased in those children whose mothers participated in the program, which suggest a decrease in the consumption of fluoride. Education is a basic strategy for fluorosis prevention and the educational intervention applied in the study group showed good participation among mothers living in a high-fluoride water area and low income.
